# Supplementary material for: Chrysanthemum morifolium Extract Ameliorates Doxorubicin-Induced Cardiotoxicity by Decreasing Apoptosis
Source: Cancers (Basel). 2022 Jan 28;14(3):683. doi: 10.3390/cancers14030683 (PMC8833354; doi:10.3390/cancers14030683)
Supplement: Supplementary file 1 [file cancers-14-00683-s001.zip › cancers-1552066-supplementary.pdf]

Supplementary Materials

1.1. Histological Analysis

The hearts were cut into two transverse slices at the mid-level of the papillary muscles. The heart tissues were fixed in 10% formalin, embedded in paraffin, sliced into 5-μm thick sections, and stained with hematoxylin eosin (HE) and picrosirius red (PSR) as described previously [20]. The sections were de-paraffinized and incubated with PSR (1.2% picric acid [Wako, Japan], 0.1% Direct Red 80 Plus (Sigma-Aldrich, Japan), 0.1% Fast Green FCF (Sigma-Aldrich, Japan) for 60 min while being protected from light. Quantitative assessments of cross-sectional myocardial cell diameter and perivascular fibrosis area were carried out as previously described [20]. HE-stained and PSR-stained sections were photographed using an Eclipse 80i microscope (Nikon, Japan). At least 50 myocardial cells with a nucleus were evaluated, and their diameter was measured using ImageJ software. Areas of perivascular fibrosis were measured using ImageJ software, and the resulting value divided by the area of the intramyocardial coronary artery was regarded as the relative vascularized fibrosis area. The scale of the measured intramyocardial coronary artery was more than 10 μm in each mouse.

1.2. Statistics

Values are shown as mean ± SEM. The Tukey-Kramer test was used to determine significant differences. A p value of < 0.05 was considered statistically significant.

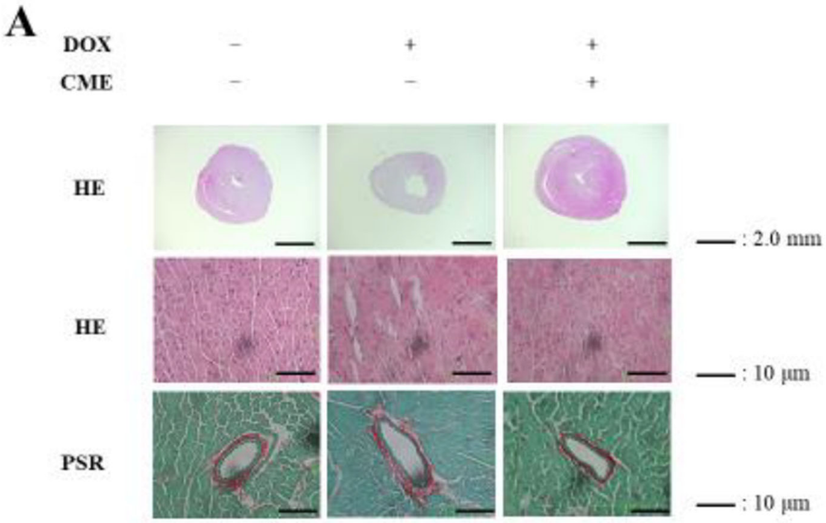

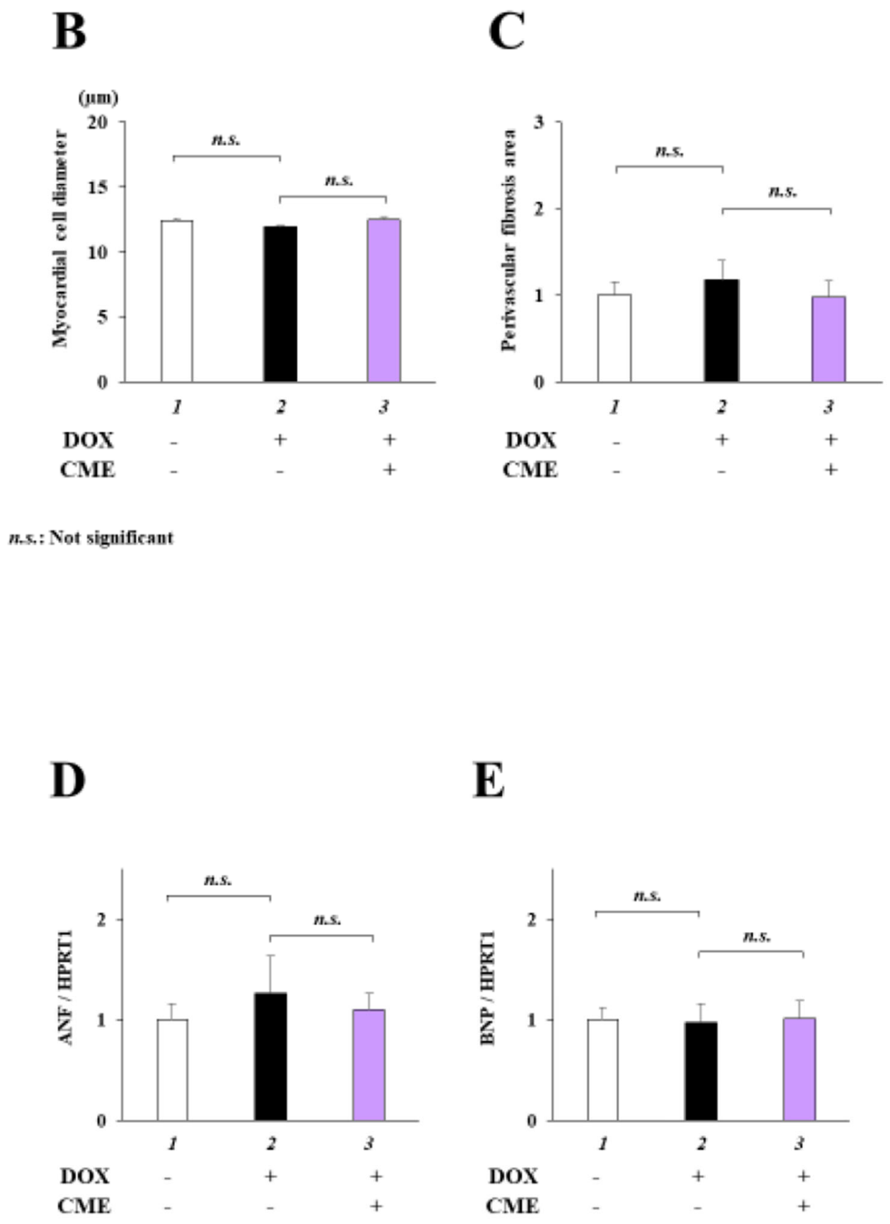

Figure S1. Representative myocardial cross-sectional images stained with hemotox-in/eosin and picrosirius red and results of quantitative RT-PCR. (**A**) Representative images of HE-stained sections of cardiomyocytes and PSR-stained perivascular fibrosis area of LV myocardium from mice. (**B**) Myocardial cell diameter was measured for at least 50 cells in each mouse. Data are presented as the mean ± SEM. (**C**) Areas of perivascular fibrosis in the left ventricle were measured for at least three intramyocardial coronary arteries with a lumen size > 10μm. Data are presented as the mean ± SEM. (**D**,**E**) Quantitative RT-PCR data for ANF (**D**), BNP (**E**), and HPRT1. Data are presented as the mean ± SEM.

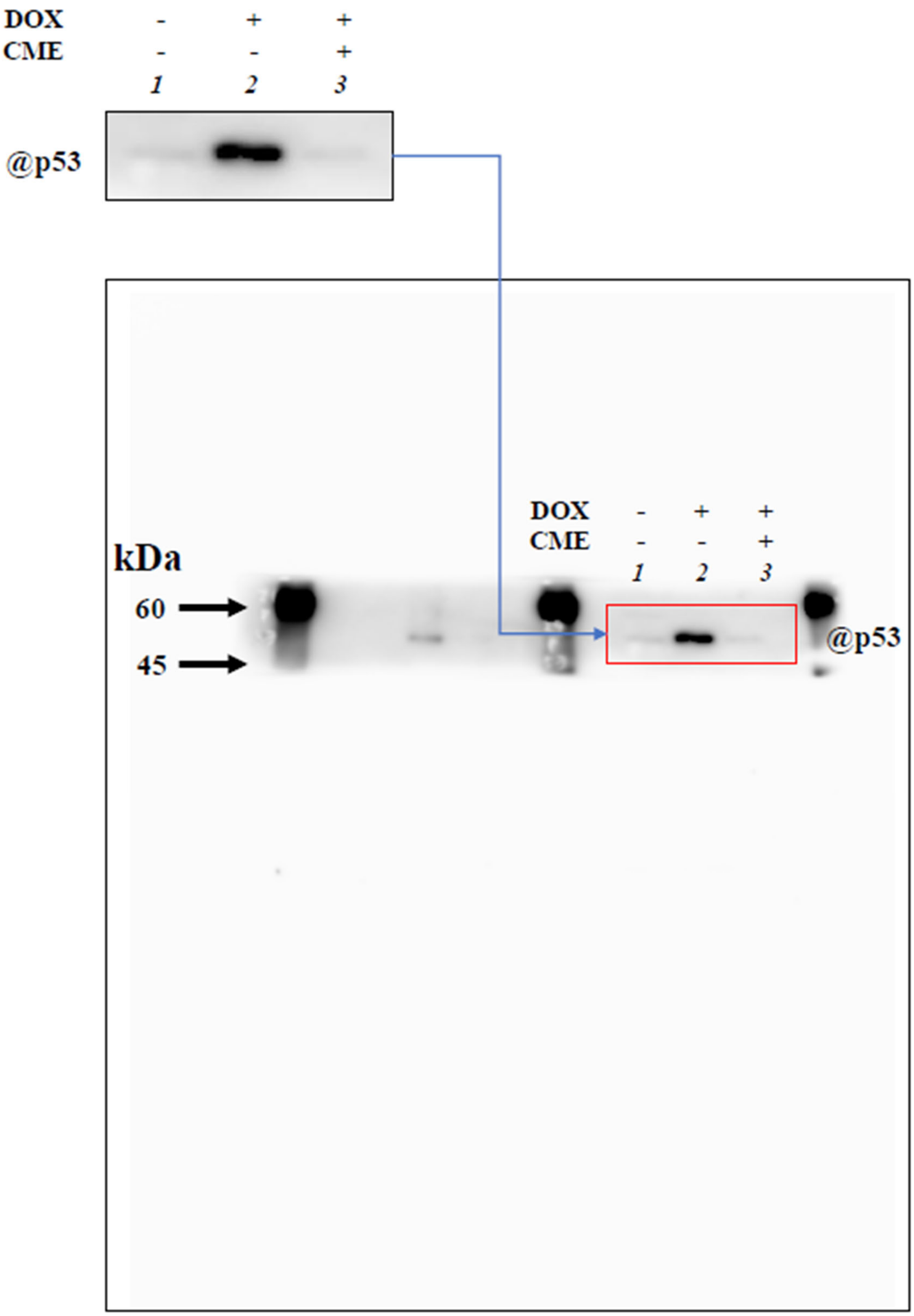
DOX - + +
CME - - +
1 2 3
@p53
kDa
60
45
DOX - + +
CME - - +
1 2 3
@p53

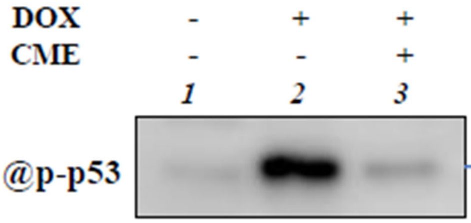
DOX - + +
CME - - +
1 2 3
@p-p53

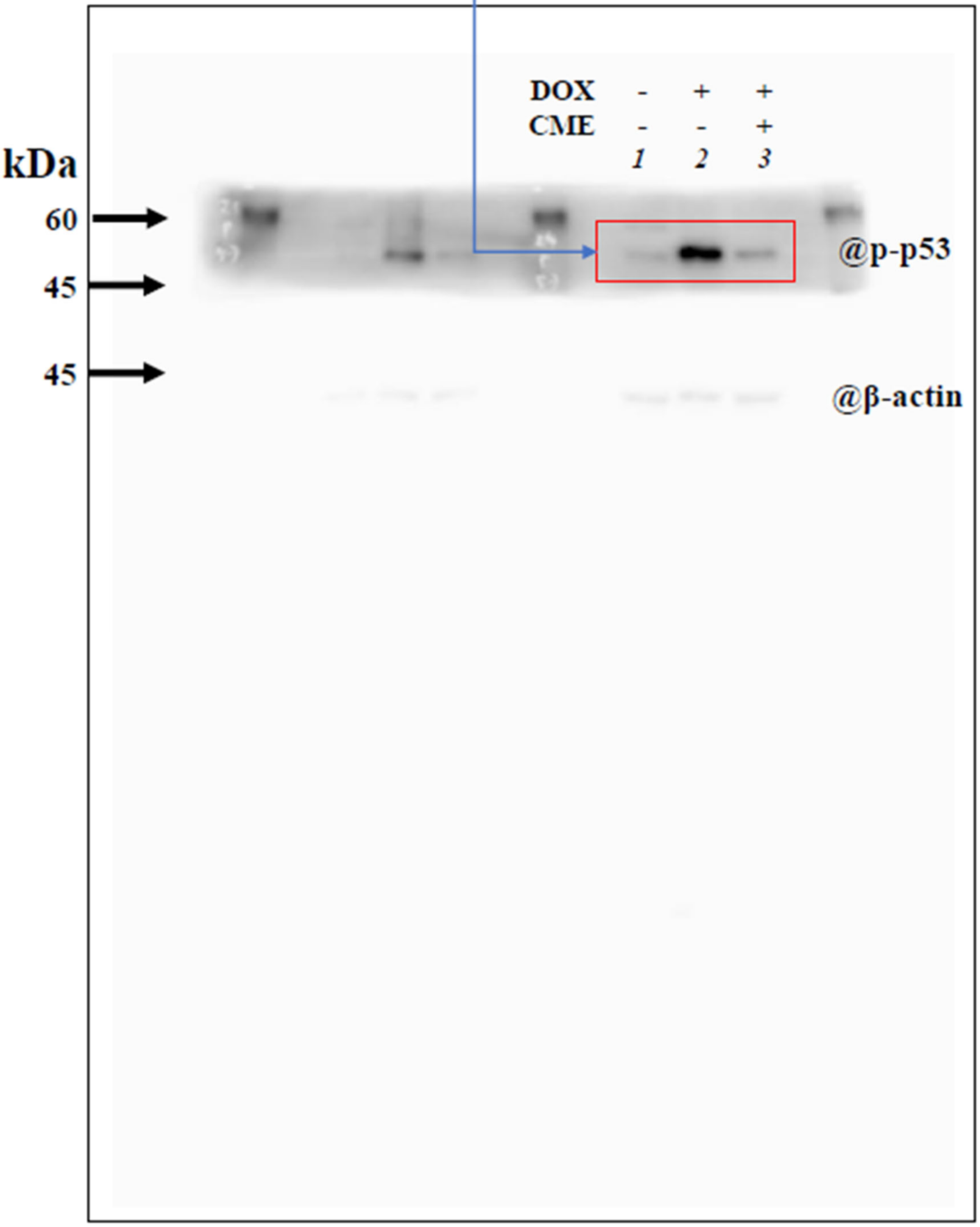
DOX - + +
CME - - +
1 2 3
kDa
60
45
45
@p-p53
@β-actin

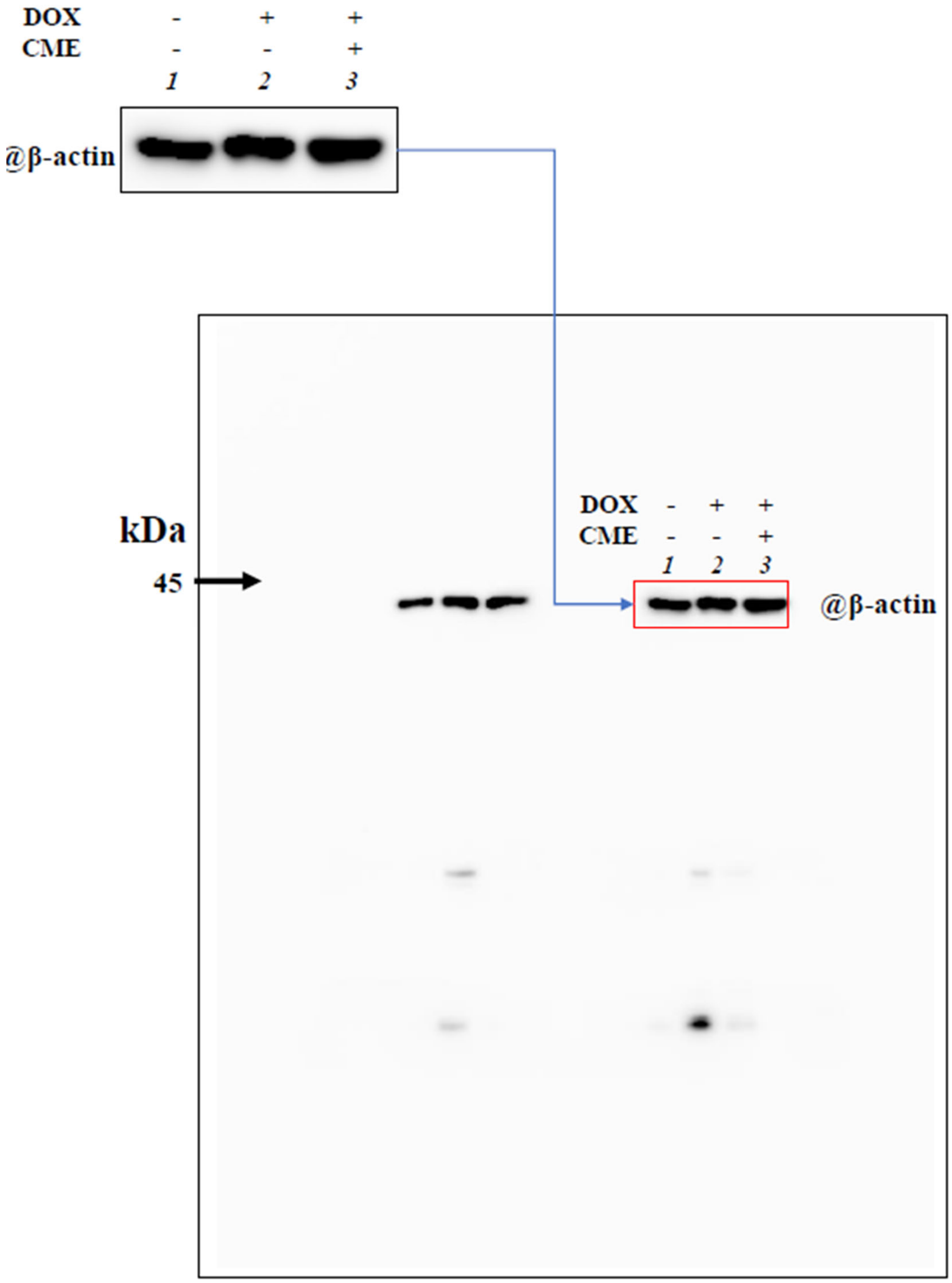

Figure S2. Original Western blots of figure 3A.

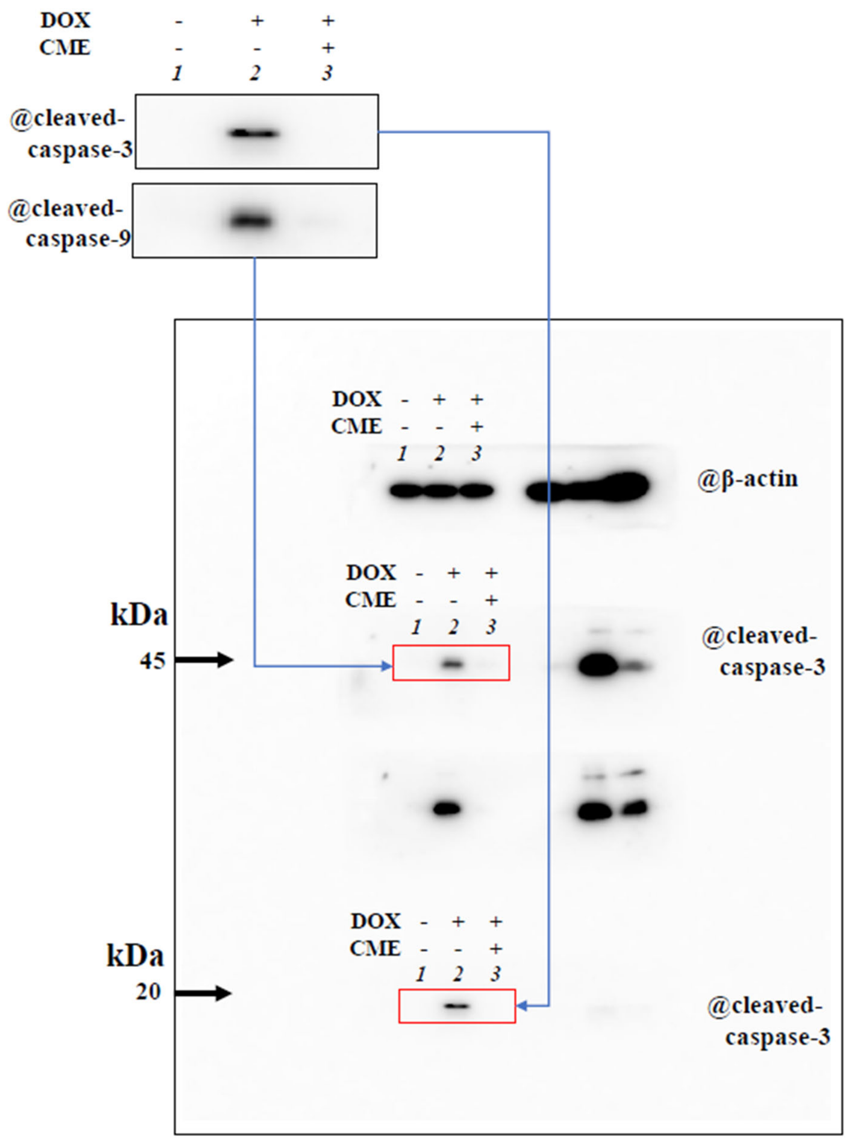
DOX - + +
CME - - +
1 2 3
@cleaved-caspase-3
@cleaved-caspase-9
DOX - + +
CME - - +
1 2 3
@β-actin
kDa
45
DOX - + +
CME - - +
1 2 3
@cleaved-caspase-3
kDa
20
DOX - + +
CME - - +
1 2 3
@cleaved-caspase-3

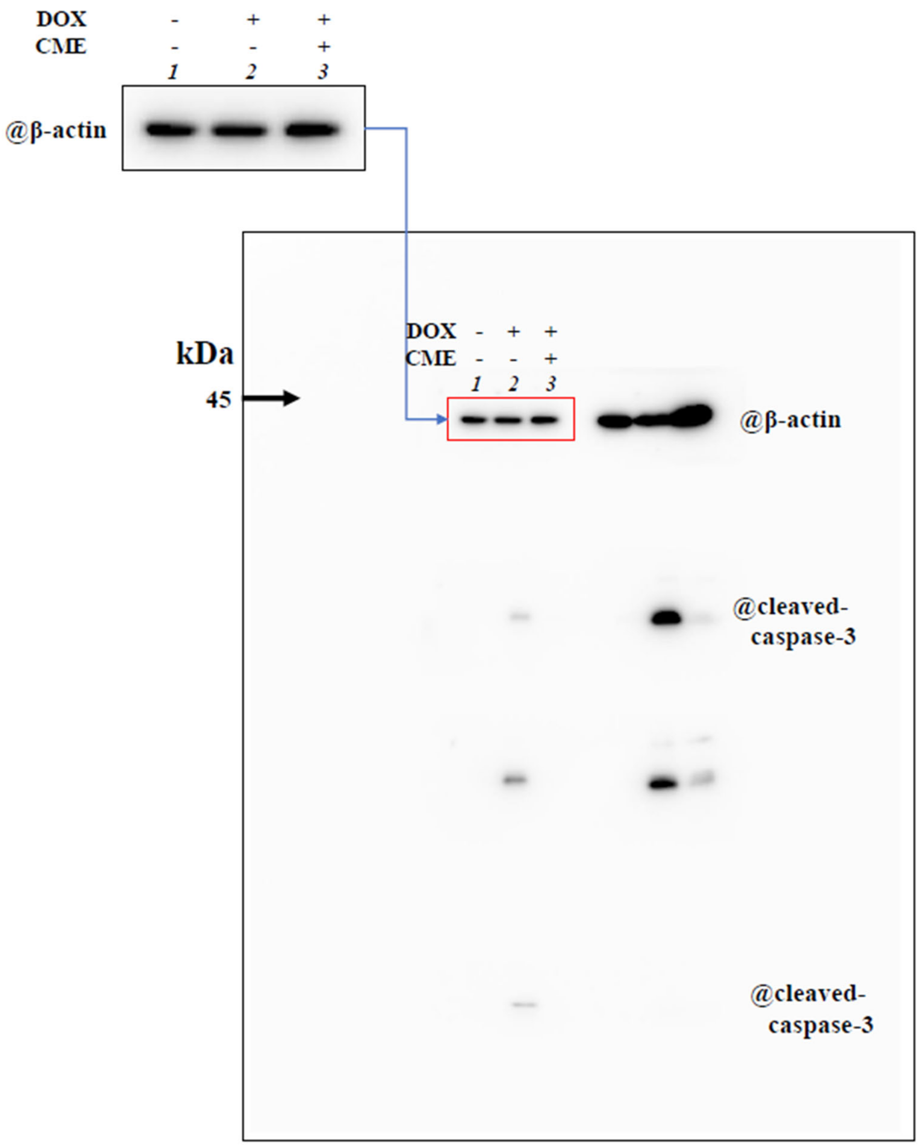

Figure S3. Original Western blots of figure 3D.
